# Exposure of Young Mice to Atmospherically Relevant PM_2.5_ Has Sex-Dependent Long-Lasting Impacts on the Skeletal Muscle System

**DOI:** 10.14336/AD.2024.1047

**Published:** 2025-12-02

**Authors:** Wenduo Liu, Zilin Wang, Min-Hye Kim, Yu Gu, Hyun-Jaung Sim, Jeong-Chae Lee, Sung-Ho Kook, Sang Hyun Kim

**Affiliations:** ^1^Department of Sports Science, College of Natural Science, Jeonbuk National University, Jeonju 54896, Korea.; ^2^Department of Bioactive Material Sciences, Research Center of Bioactive Materials, Jeonbuk National University, Jeonju 54896, Korea.; ^3^Cluster for Craniofacial Development and Regeneration Research, Institute of Oral Biosciences and School of Dentistry, Jeonbuk National University, Jeonju 54896, Korea.

**Keywords:** Particulate matter, Skeletal muscle, Mitochondria, Sex, Oxidative stress

## Abstract

The negative effects of particulate matter up to 2.5 µm in diameter (PM_2.5_) and their mediating mechanisms have been studied in various tissues. However, little is known about the mechanism and long-term tracking underlying the sex-dependent effects of PM_2.5_ on skeletal muscle system modulation. During youth, skeletal muscle grows rapidly and develops at its highest rate. Here we explore how exposure to atmospherically relevant levels of artificial PM_2.5_ affects the skeletal muscle system in 4-week-old C57BL6 mice according to sex and track the effects for 15 months post-exposure. We found that PM_2.5_ retarded muscle fiber growth and caused mitochondrial damage by modulating factors related to mitochondrial kinetics. However, the effects of PM_2.5_ on the modulation of the skeletal muscle system differed by sex and post-exposure time. The negative impacts of PM_2.5_ on skeletal muscle continued until they were overwhelmed by aging-related oxidative stress and inflammation, which were more severe in older PM_2.5_-exposed female mice compared with male mice. Older PM_2.5_-exposed female mice, but not older PM_2.5_-exposed male mice, exhibited obesity-related phenotypes in the form of increased weight and fat mass. Overall, initial exposure to PM_2.5_ affected the skeletal muscle system with long-lasting impacts that differed according to sex.

## INTRODUCTION

Industrial development and expansion have reportedly increased levels of airborne fine particulate matter (PM), which is becoming an inescapable health factor [1]. Among PM classified by diameter size, those smaller than 2.5 μm in diameter (PM_2.5_) can have negative effects on air quality and human health due to an ability to penetrate the peripheral blood system and spread throughout the body, whereas larger particulates such as PM_10_ (between 2.5 and 10 μm) do not have this ability [2, 3]. Key PM_2.5_ components include heavy metals, polycyclic aromatic hydrocarbons, and organic carbon compounds, all of which can contribute to oxidative stress, inflammation, and metabolic dysfunction [4]. The adverse effects of PM_2.5_ have been studied extensively in the lungs [5], heart [6], brain [7], fat [8], blood and bone marrow [9], leading to tissue-specific disorders. Although short-term exposure to PM_2.5_ reportedly causes acute mitochondrial damage in skeletal muscles [10], the long-term effects of PM_2.5_ on the skeletal muscle remain unexplored. Additionally, PM_2.5_-related skeletal muscle modulation during aging and according to genders have not been adequately investigated.

Skeletal muscle comprises approximately 30%-40% of total body weight [11]. As the largest metabolic organ [12] and a critical site for energy metabolism homeostasis, skeletal muscle health is integral to preventing and managing metabolic disease such as obesity, diabetes, and fatty liver disease [13-16]. At the molecular level, mitochondrial function within skeletal muscle is closely linked to these diseases, and mitochondrial biogenesis and homeostasis are essential for maintaining muscle health and metabolic function. Mitochondrial biogenesis not only enhances skeletal muscle function, but also supports muscle fiber growth, endurance, and repair [17]. However, chronic PM_2.5_ exposure is known to interfere with mitochondrial dynamics, oxidative phosphorylation, and reactive oxygen species (ROS) balance, factors that are crucial for sustaining skeletal muscle homeostasis [18].

PM_2.5_ exposure can impact mitochondrial health through several mechanisms, including oxidative stress and inflammatory signaling pathways, both of which compromise mitochondrial integrity and muscle function [10]. This study does not directly investigate these molecular mechanisms, as the primary aim is to evaluate long-term structural and functional impacts of PM_2.5_ exposure on skeletal muscle, with mechanisms to be explored in future research. Moreover, skeletal muscle, as a paracrine and endocrine organ, can crosstalk with adipose tissue and the liver, pancreas, cardiovascular system, brain, and skeleton through myokine secretion [19], establishing skeletal muscle health as crucial to overall systemic health.

The extent of skeletal muscle growth and development profoundly impacts health maintenance during aging. Skeletal muscle growth during youth rapidly increases muscle size and function, providing a foundation for resilience against age-related muscle weakness and metabolic decline [20-22]. In addition, gender differences influenced by sex hormones result in variations in skeletal muscle fiber type [23], metabolic function [24], and response to stress and aging [25]. Testosterone in males promotes muscle hypertrophy and supports a higher oxidative capacity, while estrogen in females influences muscle regeneration and metabolism through the regulation of proteins such as AMP-activated protein kinase (AMPK) and peroxisome proliferator-activated receptor gamma coactivator 1-alpha (PGC-1α), which play roles in muscle endurance and mitochondrial health [26, 27]. These hormonal effects lead to gender-specific responses to environmental stressors, including PM_2.5_ exposure.

In this study, we investigated the effects of exposure to atmospherically relevant levels of PM_2.5_ on the modulation of the skeletal muscle system according to gender in young mice for 5 consecutive days, starting at 4 weeks of age, and tracked the exposure effects for 15 months post-exposure. This approach aims to clarify sex-based differences in the physiological impacts of PM_2.5_ on skeletal muscle, providing insights for future studies on molecular mechanisms involved in these processes.

## MATERIALS AND METHODS

### Experimental animals

We purchased 3-week-old male C57BL/6 J mice and tested them after a 1-week acclimation period. The room was maintained at a controlled temperature (18-22 °C) and humidity (40%-60%) on a 12-12-h light-dark cycle. Water and feed were provided ad libitum during the breeding period. A total of 72 mice was randomly divided into four groups, and after PM_2.5_ exposure, six mice from each group were sacrificed at 1, 3, and 15 months for analysis. An overview of the experimental design is provided as Supplementary Figure 1. All experimental procedures were conducted in accordance with guidelines and regulations set by the Institutional Animal Care and Use Committee of Jeonbuk National University (CBNU-2023-114).

### Atmospheric simulation chamber

The atmospheric simulation chamber (ASC) is a whole-body exposure device designed to replicate the inhalation of PM_2.5_ from the atmosphere while maintaining a consistent average concentration, and it was used as in previous studies [8, 9, 28]. A PM solution was formulated by mixing 10 organic and inorganic compounds, including oxalic acid, malonic acid, glutaric acid, sucrose, 2,5-dihydroxybenzoic acid, glycine, ammonium sulfate, ammonium nitrate, acetate, and glycerol, in distilled water (Supplementary Table 1). The PM was aerosolized using a TQ-50C0,5 nebulizer (Meinhard, USA) and, subsequent to passing through a polypropylene melt-blown filter, particles larger than 2.5 μm in diameter were filtered into the chamber. The concentration of PM_2.5_ within the chamber was monitored continuously in real-time using a BT-610 particle counter (Met One, USA), with the predetermined concentration automatically maintained by a flow controller program. The treated mice were exposed to PM_2.5_ for 2 h per day, 5 times a week, at a concentration of 50.9 ± 10.4 μg/m^3^ (Supplementary Table 2). Chamber humidity was kept at 55%-60% within a temperature range of 23-25 °C.

### Atmospherically relevant PM2.5 chemical composition

The organic components and inorganic salts used to prepare the artificial PM_2.5_ are listed in Supplementary Table 1. Eight organic compounds with carboxylic acid, polyol, sugar, aromatic, and amino acid functional groups were investigated. Ammonium sulfate and ammonium nitrate were used as model inorganic salts because of their abundance in air [29-32]. The 10 compounds were mixed at an organic-to-inorganic dry-mass ratio of 1:1 to mimic the chemical complexity of atmospheric aerosols. The compounds were purchased from Sigma-Aldrich (purity ≥ 98%) and used without further purification. The mixture of 10 components was dissolved in purified water.

### Adipose tissue mass analysis

At the time of collection of epididymal fat and retroperitoneal adipose tissue, tissue mass was measured using an electronic balance (Sartorius Lab Instruments GmbH & Co. KG, 37070 Goettingen, Germany) to confirm changes in fat mass [33].

### Thiobarbituric acid-reactive substances

Thiobarbituric acid-reactive substances (TBARs), which are oxidative stress indicators, were analyzed after storing isolated gastrocnemius muscle (GAS) at -80 °C until analysis. Freeze-clamped muscle samples were homogenized in an ice-cold buffer (50 mM sodium phosphate, pH 7.0) and centrifuged (13,000 *g*, 10 min, 4 °C) to separate the supernatant. The supernatant was analyzed according to the manual for a Quantichrom TBARS Assay Kit (Bioassay Systems, Hayward, CA, USA) [34].

### Western blot analysis

Analysis of protein expression was conducted using a GAS that was rapidly frozen in liquid nitrogen upon extraction and stored at -80 °C. The GAS samples were homogenized in a cold buffer (50 mM Tris·HCl at pH 7.4, 1% NP-40, 0.25% sodium deoxycholate, 150 mM NaCl, 1 mM ethylenediaminetetraacetic acid at pH 7.4, 1 mM Pefabloc [Roche, Basel, Switzerland], 1 mM NaF, 1 μg/mL aprotinin, 1 μg/mL leupeptin, 1 μg/mL pepstatin, 0.1 mM bpV[phen], and 2 mg/mL β-glycerophosphate) and kept on ice. The homogenate was solubilized in a Laemmli sample buffer after determining the protein concentration using a Bradford Bio-Rad protein assay dye reagent concentrate (BIO-RAD, CA, USA) [35]. Following gel electrophoresis, each sample was transferred onto nitrocellulose membranes and blocked with skim milk at room temperature. The membranes were then incubated overnight at 4 °C with primary antibodies (peroxisome proliferator-activated receptor gamma coactivator 1-alpha [PGC-1α; GeneTex, GTX37356, CA, USA]. OXPHOS [Abcam, ab110413, USA], OXPHOS complex Ⅳ subunit Ⅳ [Invitrogen, A21348, USA], mitofusin 1 [MFN1, SCBT, sc-166644, USA], MFN2 [SCBT, sc-515647, USA], optic atrophy 1 [OPA1; SCBT, sc-393296, USA]; mitochondria fission 1 protein [Fis1; SCBT, sc-376447, USA], dynamin-related protein 1 [Drp1; SCBT, sc-32898, USA], Pax-7 [SCBT, sc-81648, USA]; myogenin [SCBT, sc-52903, USA], MuRF-1 [SCBT, sc-398608, USA], MAFbx [SCBT, sc-166806, USA], myostatin [SCBT, sc-134345, USA]; nuclear factor kappa β [NFκB; SCBT, sc-8008, USA]; adipoR1 ([CBT, sc-99183, USA], CD36 [SCBT, sc-7309, USA], PPAR-α [SCBT, sc-9000, USA], acetyl-CoA carboxylase [ACC; #3662, Cell Signal Technology], phospho-acetyl-CoA carboxylase [p-ACC; #3661, Cell Signal Technology], CPTI [SCBT, sc-393070, USA], ACADL/LCAD [Abcam, ab129711, USA], GLUT4 [SCBT, sc-53566, USA], hexokinase 2 [HK2; SCBT, sc-374091, USA], phosphofrucokinase-1 [PFK-1; SCBT, sc-166722, USA], superoxide dismutase type 1 [SOD1; SCBT, sc-8637, USA], ], superoxide dismutase type 2 [SOD2; SCBT, sc-18503, USA], catalase [SCBT, sc-271358, USA], glutathione peroxidase 4 [GPX4; SCBT, sc-166570, USA], Bax [SCBT, sc-7480, USA], Bcl2 [SCBT, sc-7382, USA], and β-actin [MA1-140 Invitrogen, MN, USA]), and then subjected to further incubation with appropriate secondary antibodies (mouse anti-goat [sc-2354, Santa Cruz Biotechnology], mouse anti-rabbit [sc-2357, Santa Cruz Biotechnology], and goat anti-mouse [sc-2005, Santa Cruz Biotechnology]) for protein detection. Protein visualization was conducted using the ECL Western Blotting Detection Reagent (GE Healthcare, Chalfont St Giles, UK), and quantification used a ChemiDoc XRS+ system (BIO-RAD, Hercules, CA, USA).

### Transmission electron microscopy

Transmission electron microscopy (TEM) was used to examine the mitochondrial structure [10]. The soleus muscle was fixed in a solution containing 2.5% glutaraldehyde and 4% formaldehyde and a 0.1 M phosphate buffer at pH 7.4 for 2 h immediately after extraction. The fixed muscles were post-fixed with 1% osmium tetroxide for 2 h. The muscles were then dehydrated using a graded series of ethanol solutions and embedded in Epon-812 resin. Sections were obtained using a NOVA ultramicrotome (LKB, Vienna, Austria) and mounted on a 100-mesh grid. Sections approximately 80 nm thick were prepared for TEM and stained with 0.1% toluidine blue. To enhance visualization, the sections were stained with uranyl acetate and lead citrate and examined using an H7650 electron microscope (Hitachi, Japan) with an accelerating voltage of 80 kV to confirm the specimens. Samples were analyzed using a JEM-2010 TEM (JEOL) at the Center for University Wide Research Facilities at Jeonbuk National University.

### Hematoxylin and eosin staining

Histologic analysis of muscle samples (Solues) was performed according to a previously developed protocol [33]. Tissues were fixed using formalin at 4 °C. After dehydration with ethanol, each tissue was clarified, infiltrated, and embedded in paraffin with xylene. The embedded tissues were sectioned and stained with hematoxylin and eosin (H&E) on glass slides. The size of muscle fibers was measured using a MoticEasyScan One slide scanner (Meyer Instruments, Inc., Houston, TX, USA) and further quantified in ImageJ software (version 1.51, NIH, Bethesda, MD, USA).

**Figure 1.**
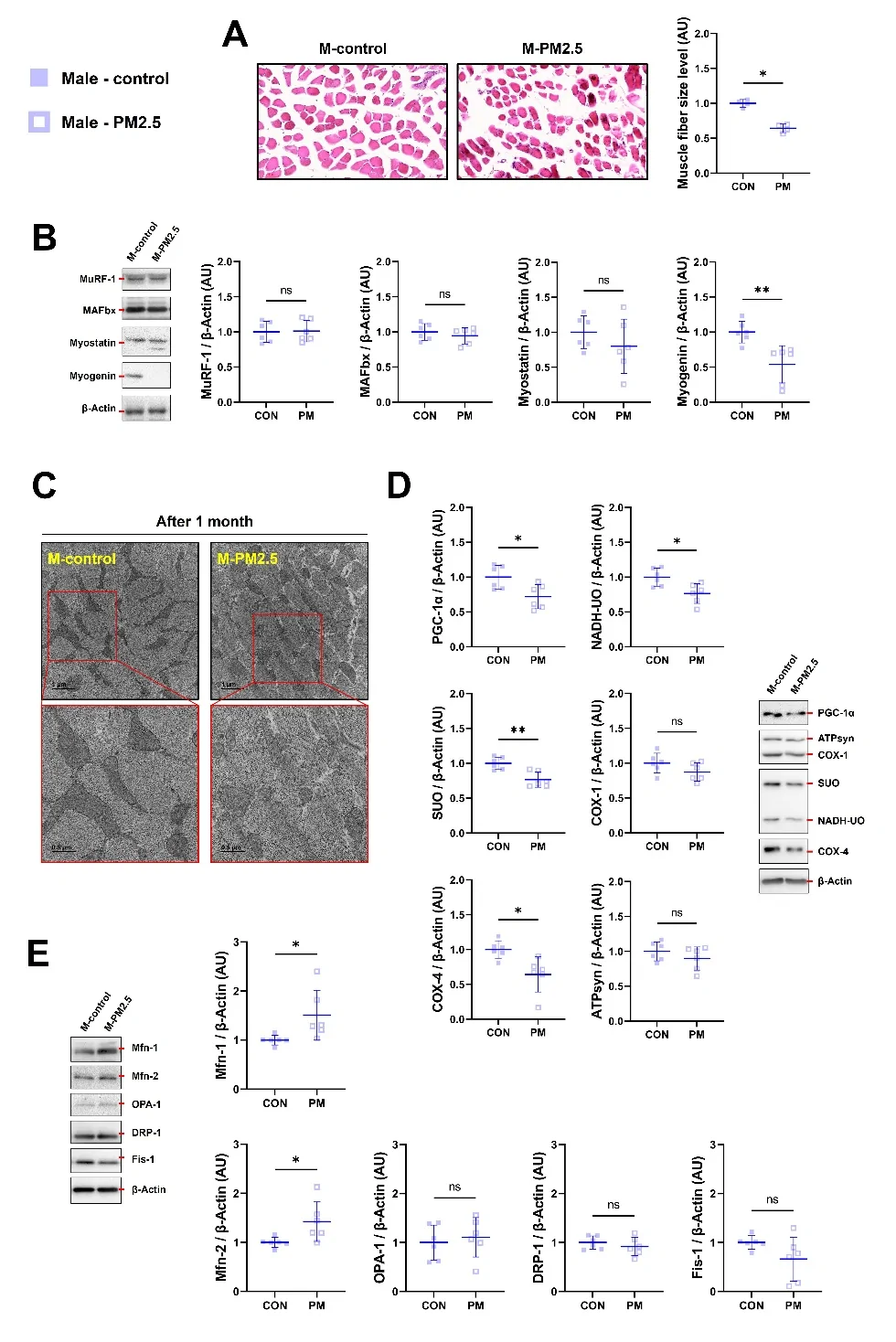
**Effect of PM_2.5_ on development of skeletal muscle fibers and mitochondria at 1-month post-exposure in male mice**. (**A**) Representative H&E staining imagery and muscle fiber size in soleus muscle; representative data are shown (n = 4). (**B**) Expression levels of MuRF-1, MAFbx, myostatin, and Myogenin proteins in gastrocnemius muscles; representative data are shown (n = 6). (**C**) Representative transmission electron microscope image of soleus muscle. (**D**) Expression levels of PGC-1α, NADH-UO, SUO, COX-1, COX-4, and ATPsyn in gastrocnemius muscle; representative data are shown (n = 6). (**E**) Expression levels of Mfn-1, Mfn-2, OPA-1, DRP-1, and Fis-1 in gastrocnemius muscle; representative data are shown (n = 6). Data are presented as mean ± standard deviation. Data were analyzed using two-sided unpaired Student’s t-tests or non-parametric test (*p < 0.05; **p < 0.01; ***p < 0.001; ns, not significant, p> 0.05).

### Immunofluorescence and confocal microscopy analysis

Immunofluorescence analysis was performed according to a previously developed protocol [36]. Sections of tibialis anterior muscle samples 5 μm thick were rehydrated and permeabilized using PBS-Tween (PBS-T; 0.25% Triton X-100) for 10 min, then blocked in PBS with 1% bovine serum albumin for 30 min. Sections were incubated in a humid dark box at 4 °C overnight with the following primary antibodies: Pax-7 (SCBT, sc-81648, USA), β-galactosidase (Cell Signaling, #27198, USA), and P16 (SCBT, sc-759, USA). After three washes with PBS-T, the samples and secondary antibodies (Abcam, goat anti-rabbit, Alexa Fluor 488, ab150077; goat anti-mouse, Alexa Fluor 594, ab150116, USA) were incubated for 1 h at room temperature and protected from light. After rinsing three times with PBS-T, the tablets were sealed using a mounting medium with 4ʹ,6-diamidino-2-phenylindole (ChemCruz, sc-24941, USA). Observations were made using a super-resolution laser confocal scanning microscope and the resulting images were analyzed in Image-Pro Plus.

### Statistical analysis

All data are expressed as the mean ± standard deviation and were analyzed using GraphPad Software (Prism 9, Boston, MA, USA). Differences between two groups were analyzed by unpaired Student’s *t*-test (n ≥ 6) or by a non-parametric test (Wilcoxon *t*-test, n < 6). The Kolmogorov-Smirnov test was used to test the normality of data sets. A value of *p* <.05 was considered statistically significant.

**Figure 2.**
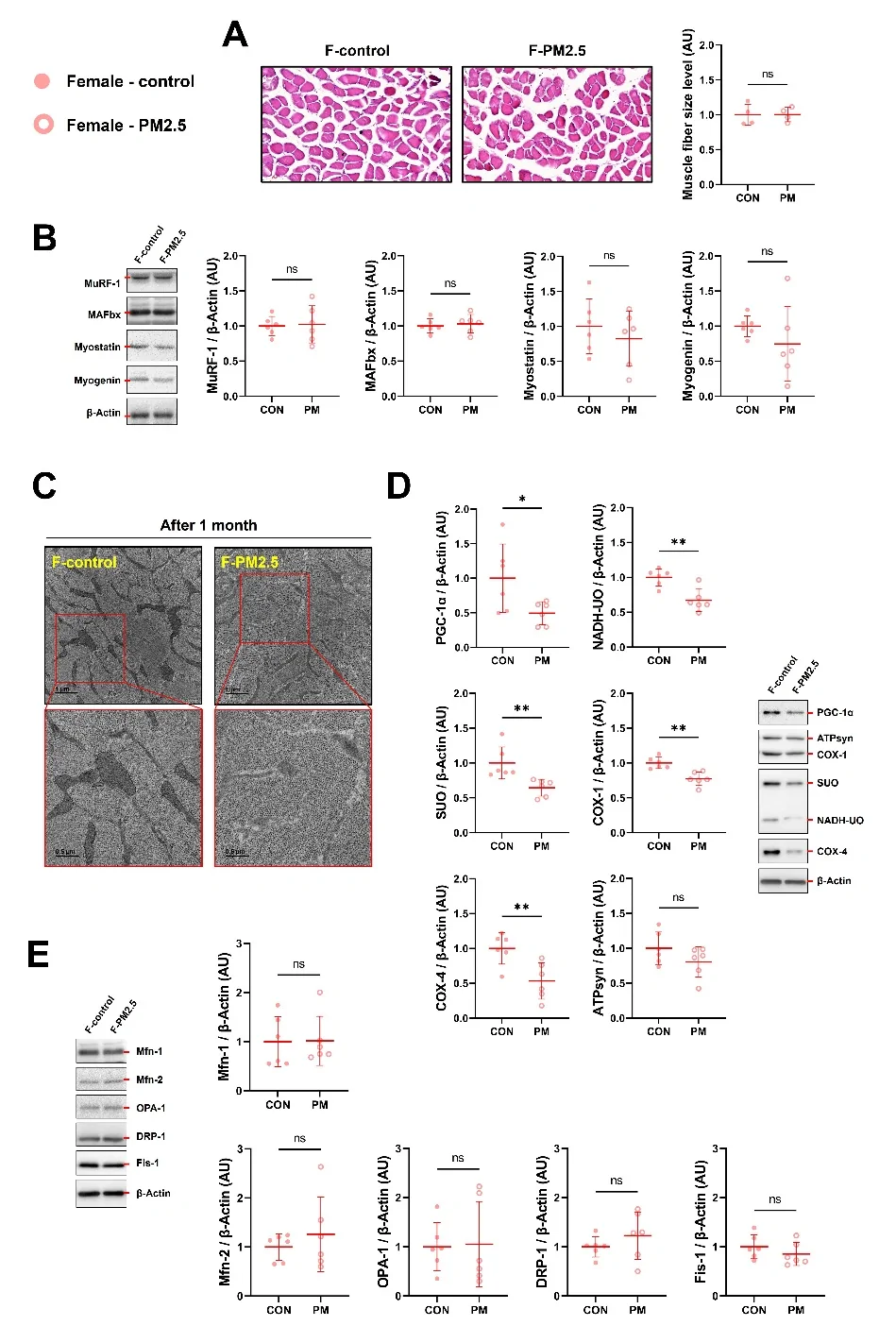
**Effect of PM_2.5_ on development of skeletal muscle fibers and mitochondria at 1-month post-exposure in female mice**. (**A**) Representative H&E staining imagery and muscle fiber size in soleus muscle; representative data are shown (n = 4). (**B**) Expression levels of MuRF-1, MAFbx, myostatin, and Myogenin in gastrocnemius muscle; representative data are shown (n = 6). (**C**) Representative transmission electron microscope image of soleus muscle. (**D**) Expression levels PGC-1α, NADH-UO, SUO, COX-1, COX-4, and ATPsyn in gastrocnemius muscle; representative data are shown (n = 6). (**E**) Expression levels of Mfn-1, Mfn-2, OPA-1, DRP-1, and Fis-1 in gastrocnemius muscle; representative data are shown (n = 6). Data are presented as mean ± standard deviation. Data were analyzed using two-sided unpaired Student’s t-tests or non-parametric test (*p < 0.05; **p < 0.01; ***p < 0.001; ns, not significant, p> 0.05).

## RESULTS

### Effect of PM_2.5_ on development of skeletal muscle fibers at 1 month post-exposure depending on sex

We examined how PM_2.5_ affected the development of muscle fibers and whether it differentially affected those of one sex over the other. We exposed mice (4 weeks old) to PM_2.5_ (at a mass concentration of approximately 50 μg/m^3^ PM_2.5_ for 2 h for 5 consecutive days) in an ASC and initially investigated the fiber muscles of the mice at 1 month post-exposure (hereafter referred as young PM_2.5_-exposed mice). Young PM_2.5_-exposed male mice were significantly smaller size and expressed less MyoG, a muscle-specific transcription factor for induction of myogenesis during muscle differentiation and development, in skeletal muscle fibers compared with corresponding control male mice (Figs. 1A and B). However, the expression levels of E3 ubiquitin ligases such as MuRF-1, MAFbx, and myostatin (which is involved in the inhibition of muscle growth), were comparable between young control and PM_2.5_-exposed male mice (Fig. 1B). It has been reported that mitochondria modulate the maintenance of muscle fiber homeostasis [37]. Analysis using TEM revealed a small amount of damage to the outer mitochondrial membrane as well as changes in the internal structure of the mitochondria in young PM_2.5_-exposed male mice (Fig. 1C). These results reflect statistically decreased levels of mitochondrial enzymes such as PGC-1α, NADH-UO, SUO, and COX-4, but not Cox1 and ATPsyn (Fig. 1D). PGC-1α is a key regulator of mitochondrial biogenesis that modulates the expression of the mitochondrial enzymes (NADH-UO, SUO, COX-4, Cox1 and ATPsyn). The simultaneous reduction of those also suggests that exposure to PM_2.5_ can cause mitochondrial damage and reduce mitochondrial biogenesis, leading to slow mitochondrial remodeling in the short term. Among mitochondria fusion-related factors (Mfn-1, Mfn-2 and OPA-1), Mfn-1 and Mfn-2 level were increased only in young PM_2.5_-exposed male mice compared with corresponding control male mice (Fig. 1E). The expression levels of mitochondrial fission-related factors (DRP-1 and Fis-1) did not show any changes in the exposed male mice (Fig. 1E).

Unlike the young PM_2.5_-exposed male mice, young PM_2.5_-exposed female mice did not show any alternation in muscle fiber size and in muscle growth-related factors such as MuRF-1, MAFbx, myostatin, and MyoG (Figs. 2A and B). However, exposed female mice displayed severe mitochondrial damage with a large collapse and had a severe disruption of the outer mitochondrial membranes (Fig. 2C). Expression levels of the mitochondrial biogenesis regulator (PGC-1α) and mitochondrial electron-transport-chain-complex enzymes (NADH-UO, SUO, COX-1, and COX-4) were significantly lower in young PM_2.5_-exposed female mice compared with control female mice (Fig. 2D). The exposed female mice exhibited no change in mitochondrial fission- and fusion-related factors compared with female control mice (Fig. 2E). These findings suggest that PM_2.5_ impairs muscle fibers by modulating mitochondria via mechanisms that differ by sex.

When analyzing the metabolic adaptation at 1 month after exposure, compared with the control group, the expression of PPAR-α, LCAD, and GLUT-4 was significantly decreased in male mice in the PM_2.5_ exposure group (Supplementary Figs. 4A and B). This indicates that mitochondrial damage is accompanied by metabolic disorders. However, among the metabolic factors in female mice, only the protein expression of GLUT-4 was significantly reduced between the PM_2.5_-exposed group and the control group, while there were no significant differences in other factors (Supplementary Figs. 4C and D).

### Effect of PM_2.5_ on restoration of skeletal muscle fibers at 3 months post-exposure depending on sex

**Figure 3.**
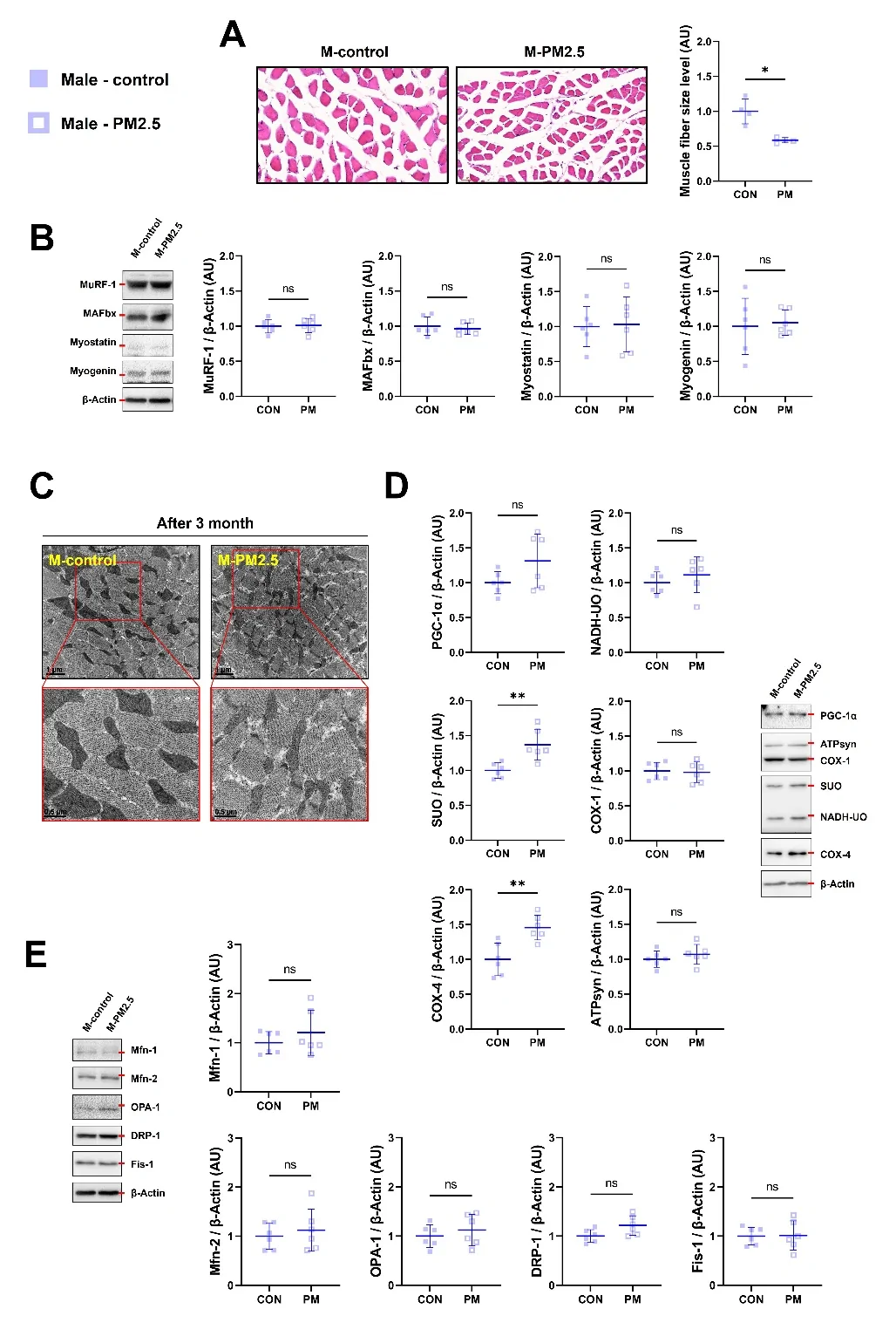
**Effect of PM_2.5_ on restoration of skeletal muscle fibers and mitochondria at 3 months post-exposure in male mice**. (**A**) Representative H&E staining imagery and muscle fiber size in soleus muscle; representative data are shown (n = 4). (**B**) Expression levels of MuRF-1, MAFbx, myostatin, and Myogenin in gastrocnemius muscle; representative data are shown (n = 6). (**C**) Representative image of a transmission electron microscope in soleus muscle. (**D**) Expression levels of PGC-1α, NADH-UO, SUO, COX-1, COX-4, and ATPsyn in gastrocnemius muscle; representative data are shown (n = 6). (**E**) Expression levels of Mfn-1, Mfn-2, OPA-1, DRP-1, and Fis-1 in gastrocnemius muscle; representative data are shown (n = 6). Data are presented as mean ± standard deviation. Data were analyzed using two-sided unpaired Student’s t-tests or non-parametric test (*p < 0.05; **p < 0.01; ***p < 0.001; ns, not significant, p>0.05).

As initial exposure to PM_2.5_ reportedly has a lasting impact on lung function [9], we questioned whether PM_2.5_-impaired fiber muscles can be restored by physiological conditioning. To answer the question, we investigated skeletal muscle fibers from adult PM_2.5_-exposed mice 3 months after the initial exposure. Damaged muscle fibers are regenerated by the propagation and differentiation of satellite stem cells via symmetric and/or asymmetric manners. The muscle fibers of adult PM_2.5_-exposed male mice were still smaller with no change in muscle growth-related factors compared with those of corresponding control male mice (Figs. 3A and B). PM_2.5_-induced mitochondrial damage was restored in the exposed male mice, which exhibited higher levels of mitochondrial electron-transport-chain-complex enzymes such as NADH-UO, SUO, and COX-4, in comparison with corresponding control male mice (Figs. 3C and D). No significant differences in the expression levels of mitochondrial fusion- and fission-related factors were observed between adult controls and PM_2.5_-exposed male mice (Fig. 3E).

**Figure 4.**
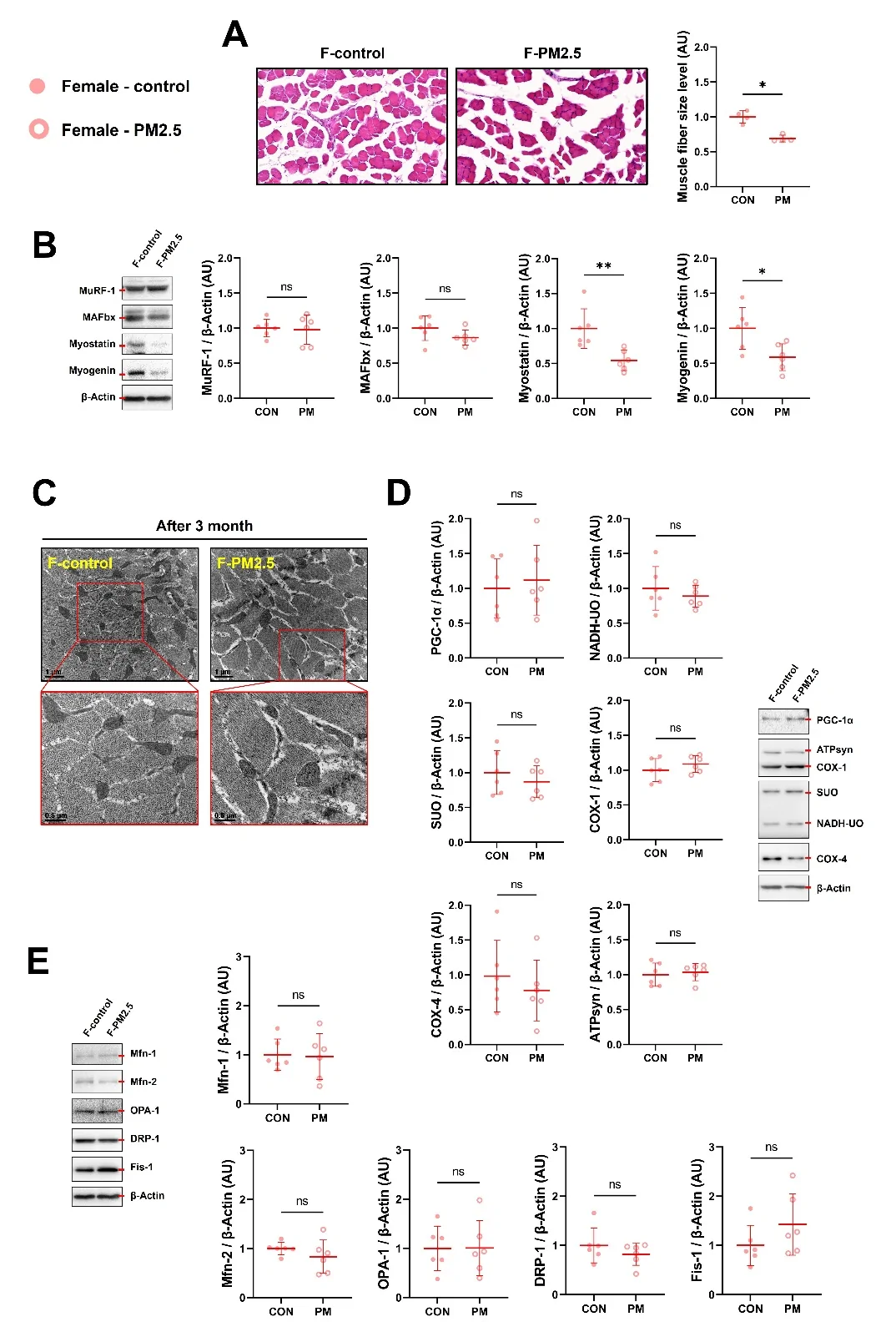
**Effect of PM_2.5_ on restoration of skeletal muscle fibers and mitochondria at 3 months post-exposure in female mice**. (**A**) Representative H&E staining imagery and muscle fiber size in soleus muscle; representative data are shown (n = 4). (**B**) Expression levels of MuRF-1, MAFbx, myostatin, and Myogenin in gastrocnemius muscle; representative data are shown (n = 6). (**C**) Representative transmission electron microscope imagery of soleus muscle. (**D**) Expression levels of PGC-1α, NADH-UO, SUO, COX-1, COX-4, and ATPsyn in gastrocnemius muscle; representative data are shown (n = 6). E) Expression levels of Mfn-1, Mfn-2, OPA-1, DRP-1, and Fis-1 in gastrocnemius muscle; representative data are shown (n = 6). Data are presented as mean ± standard deviation. Data were analyzed using two-sided unpaired Student’s t-tests or non-parametric test (*p < 0.05; **p < 0.01; ***p < 0.001; ns, not significant, p>0.05).

Adult PM_2.5_-exposed female mice, but not young PM_2.5_-exposed female mice, had significantly smaller muscle fibers compared with corresponding female control mice (Fig. 4A). This observation was associated with decreased expression of MyoD in adult PM_2.5_-exposed female mice (Fig. 4B). Similarly, expression myostatin was reduced in exposed female mice. Myostatin is a negative regulator of muscle growth and plays a role in reducing muscle fiber size. However, when expression of myostatin decreases, inhibitory signals associated with muscle growth weaken, promoting a physiological response to prevent muscle loss [38-40], implying activation of a mechanism to suppress expression of myostatin prevents muscle atrophy to compensate for inadequate recovery and regeneration of muscle cells due to MyoG reduction (Fig. 4B). PM_2.5_-exposed female mice also exhibited restoration of PM_2.5_-caused mitochondrial damage without noticeable turbulence in mitochondrial dynamic-related factors (Figs. 4C-E). Collectively, these findings suggest a discernible difference in the susceptibility and restoration potential of muscle fibers in response to exposure to PM_2.5_ depending on sex.

We investigated the recovery of muscle fibers and related metabolic adaptations at 3 months post-exposure. In female mice, PPAR-α expression remained significantly lower in the PM_2.5_ exposure group compared to the control group (Supplementary Fig. 5C), indicating an impact on lipid metabolism. However, no significant differences were observed in other markers (Supplementary Fig. 5D). Similarly, male mice showed no significant differences in metabolic factors between the PM_2.5_ exposure group and the control group (Supplementary Figs. 5A and B). This suggests that normalization occurs over time in males, while females exhibit a relatively delayed response to PM_2.5_.

### Effect of PM_2.5_ on aging of skeletal muscle fibers at 15 months post-exposure depending on sex

**Figure 5.**
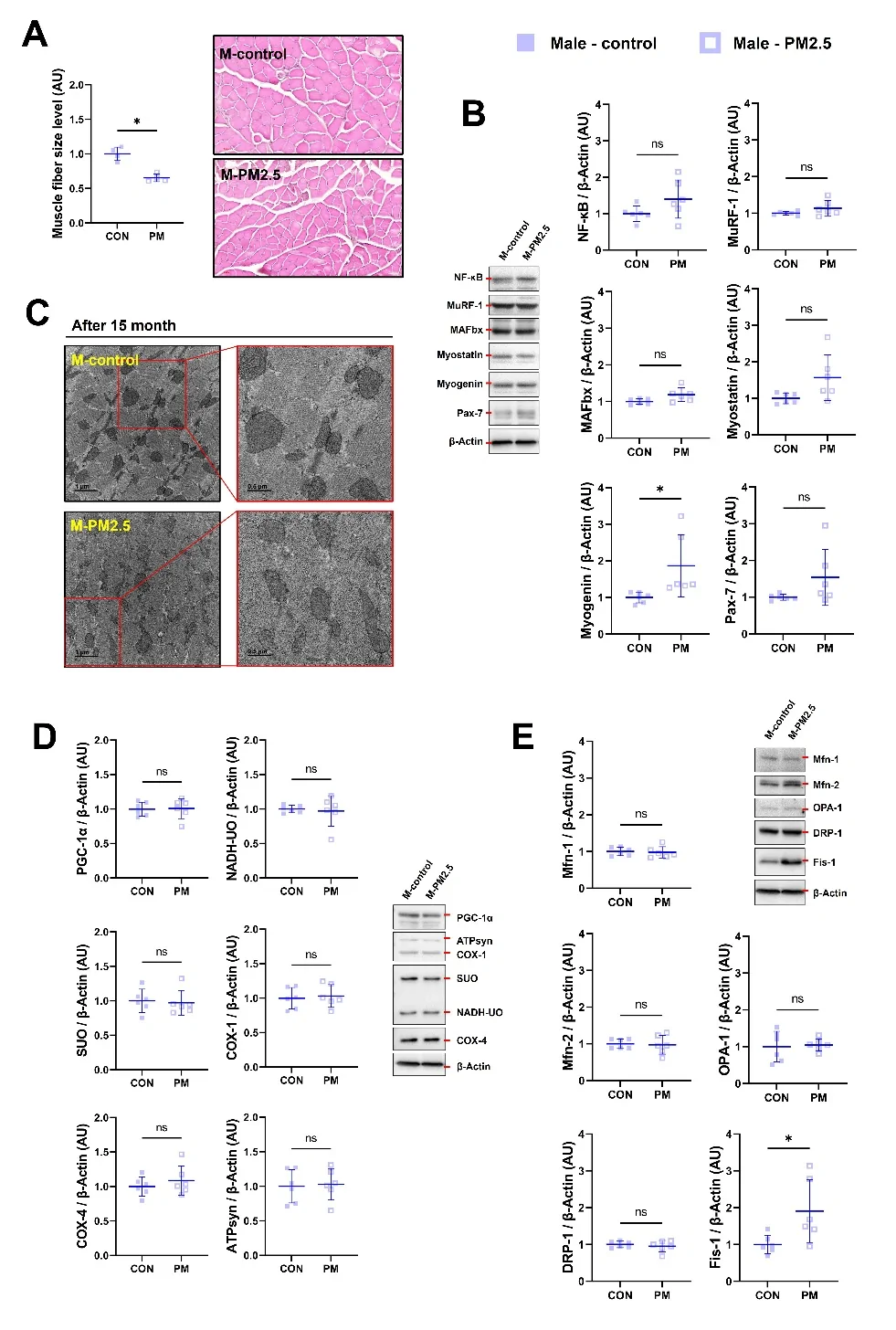
**Effect of PM_2.5_ on aging of skeletal muscle fibers and mitochondria in male mice at 15 months post-exposure**. (**A**) Representative H&E staining imagery and muscle fiber size in soleus muscle; representative data are shown (n = 4). (**B**) Expression levels of NF-κB, MuRF-1, MAFbx, myostatin, Myogenin, and Pax-7 in gastrocnemius muscle; representative data are shown (n = 6). (**C**) Representative transmission electron microscope imagery of soleus muscle. (**D**) Expression levels of PGC-1α, NADH-UO, SUO, COX-1, COX-4, and ATPsyn in gastrocnemius muscle; representative data are shown (n = 6). (**E**) Expression levels of Mfn-1, Mfn-2, OPA-1, DRP-1, and Fis-1 in gastrocnemius muscle; representative data are shown (n = 6). Data are presented as mean ± standard deviation. Data were analyzed using two-sided unpaired Student’s t-tests or non-parametric test (*p < 0.05; **p < 0.01; ***p < 0.001; ns, not significant, p>0.05).

**Figure 6.**
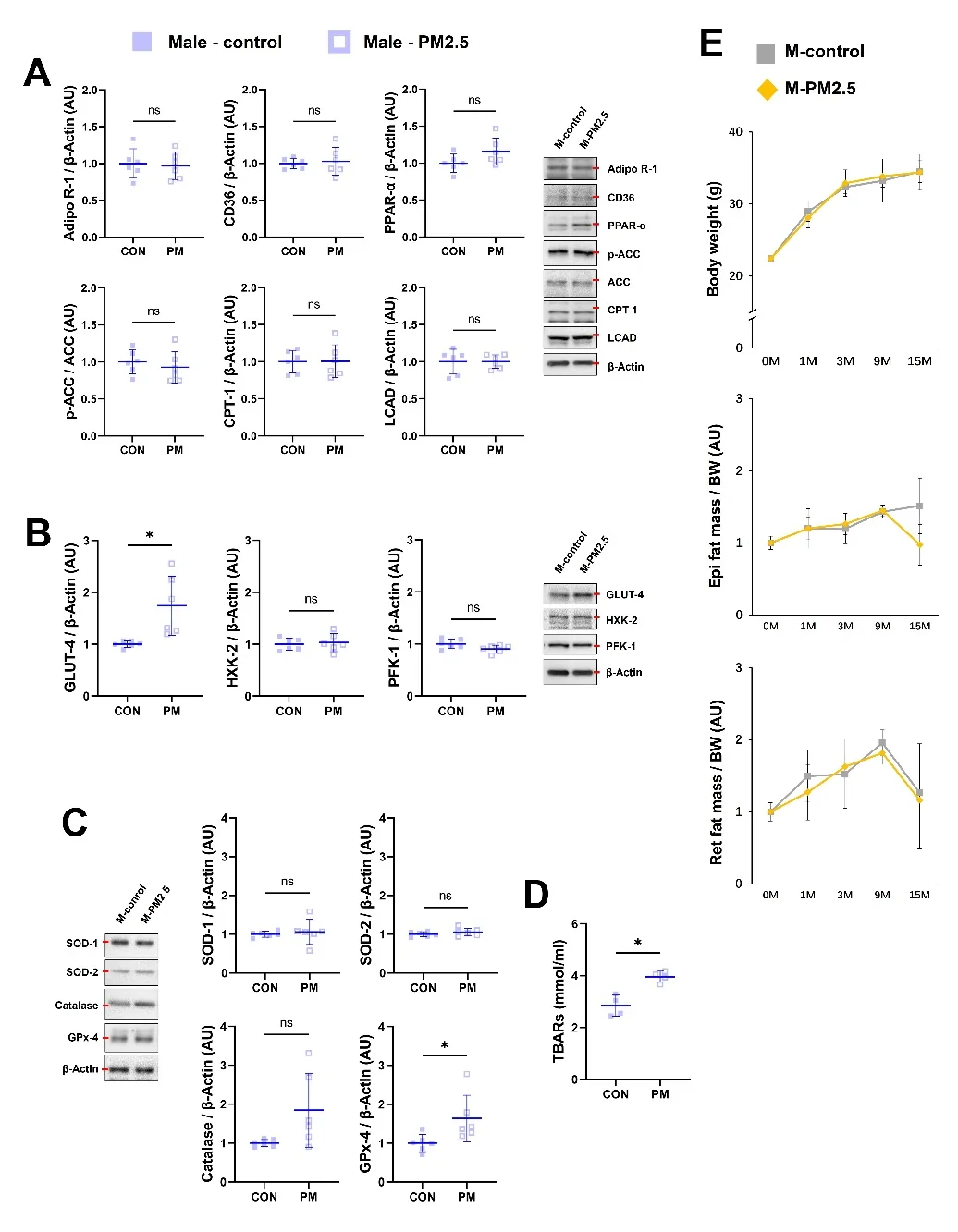
**Effect of PM_2.5_ on aging of skeletal muscle metabolism and oxidative stress in male mice at 15 months post-exposure**. (**A**) Expression levels of Adipo R-1, CD36, PPAR-α, CPT-1, LCAD, and ACC phosphorylation in gastrocnemius muscle; representative data are shown (n = 6). (**B**) Expression levels of GLUT-4, HXK-2, and PFK-1 in gastrocnemius muscle; representative data are shown (n = 6). (**C**) Expression levels of SOD-1, SOD-2, catalase, and GPx-4 in gastrocnemius muscle; representative data are shown (n = 6). (**D**) TBARs level in gastrocnemius muscle; representative data are shown (n = 4). (**E**) Changes in body weight, epididymal fat mass and retroperitoneal fat pad mass after PM_2.5_ exposure; representative data are shown (n = 6). Data are presented as mean ± standard deviation. Data were analyzed using two-sided unpaired Student’s t-tests or non-parametric test (*p < 0.05; **p < 0.01; ***p < 0.001; ns, not significant, p>0.05).

Muscle strength and endurance appear to decline with age, leading to a reduction in the number and size of muscle fibers [33]. We therefore investigated the long-term effects of PM_2.5_ in muscle fibers of PM_2.5_-exposed mice at 15 months post-exposure (hereafter old PM_2.5_-exposed mice). Long-term deterioration following initial PM_2.5_ exposure was evident in the size of muscle fibers in old male mice. As shown in Fig. 5, old PM_2.5_-exposed male mice had significantly smaller soleus muscle fibers compared with corresponding control male mice, although the former displayed increased expression level of MyoG (Figs. 5A and B). The mitochondria of aged male mice exposed to PM_2.5_ continued to recover and, with the exception of increased expression of Fis-1, mitochondrial dynamics-related factors stabilized and were comparable to those of corresponding control male mice (Figs. 5C-E). Similarly, with the exception of increased expression of GLUT-4 and GPx-4, no change in indicators of lipid metabolism (Adipo R1, CD36, PPARγ, p-ACC, CPT-1, and LCAD), glucose metabolism (HXK-2, and PFK-1), and antioxidant enzymes (SOD-1, SOD-2, and catalase) was observed in old PM_2.5_-exposed male mice in comparison with corresponding control male mice (Figs. 6A-C). However, the concentration of TBARs, which are lipid peroxidation-associated markers, was greater in the gastrocnemius muscle fibers of old PM_2.5_-exposed male mice than in corresponding male control mice (Fig. 6D). Oxidative stress may therefore be latent in muscle fibers exposed to PM_2.5_. The physiological state of old PM_2.5_-exposed males was not associated with body weight and fat mass similar to those of corresponding control male mice (Fig. 6E).

**Figure 7.**
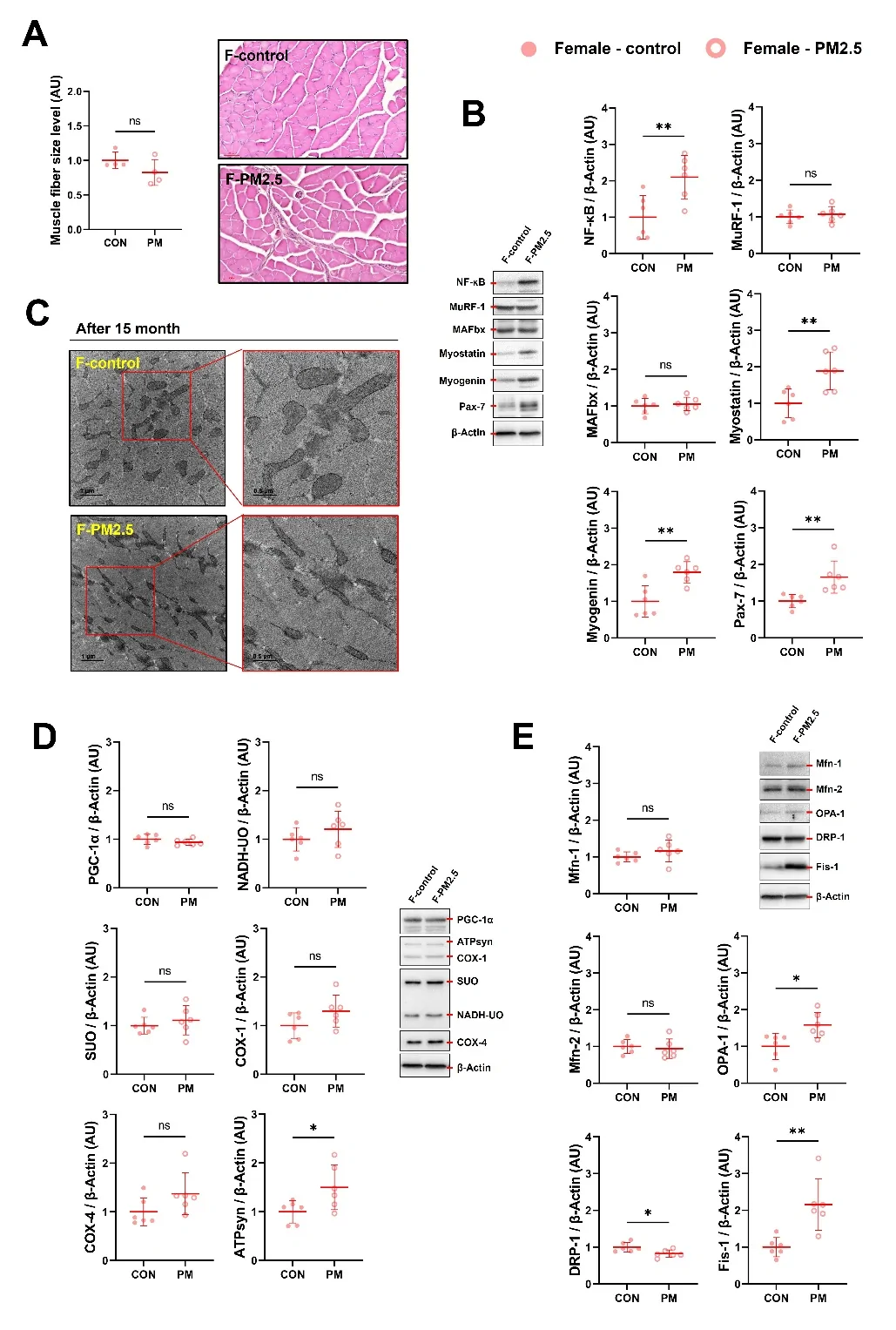
**Effect of PM_2.5_ on aging of skeletal muscle fibers and mitochondria in female mice at 15 months post-exposure**. (**A**) Representative H&E staining imagery and muscle fiber size in soleus muscle; representative data are shown (n = 4). (**B**) Expression levels of NF-κB, MuRF-1, MAFbx, myostatin, Myogenin, and Pax-7 in gastrocnemius muscle; representative data are shown (n = 6). (**C**) Representative transmission electron microscope imagery of soleus muscle. (**D**) Expression levels of PGC-1α, NADH-UO, SUO, COX-1, COX-4, and ATPsyn in gastrocnemius muscle; representative data are shown (n = 6). (**E**) Expression levels of Mfn-1, Mfn-2, OPA-1, DRP-1, and Fis-1 in gastrocnemius muscle; representative data are shown (n = 6). Data are presented as mean ± standard deviation. Data were analyzed using two-sided unpaired Student’s t-tests or non-parametric test (*p < 0.05; **p < 0.01; ***p < 0.001; ns, not significant, p>0.05).

**Figure 8.**
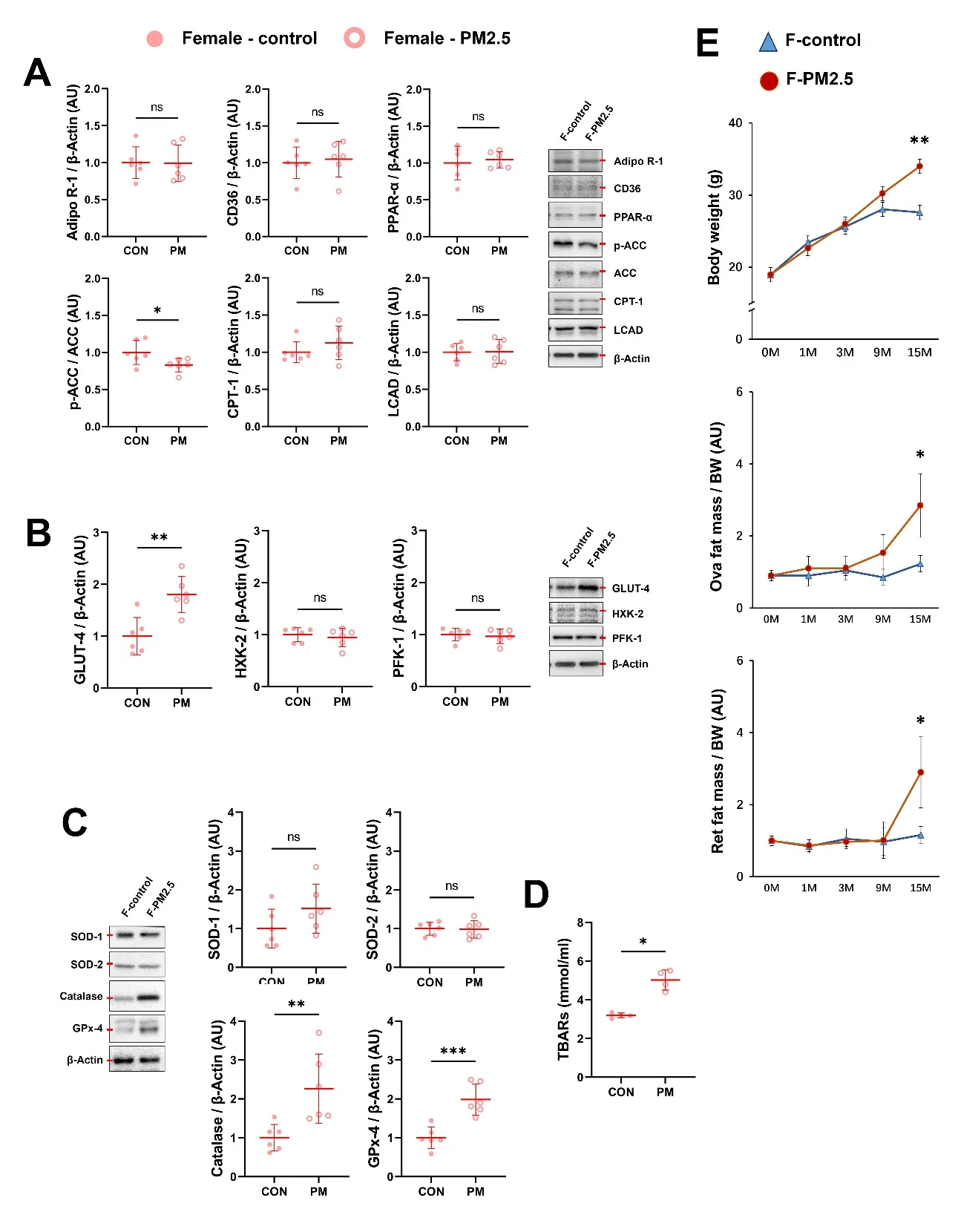
**Effect of PM_2.5_ on aging of skeletal muscle metabolism and oxidative stress in female mice at 15 months post-exposure**. (**A**) Expression levels of Adipo R-1, CD36, PPAR-α, CPT-1, LCAD, and ACC phosphorylation in gastrocnemius muscle; representative data are shown (n = 6). (**B**) Expression levels of GLUT-4, HXK-2, and PFK-1 in gastrocnemius muscle; representative data are shown (n = 6). (**C**) Expression levels of SOD-1, SOD-2, catalase, and GPx-4 in gastrocnemius muscle; representative data are shown (n = 6). (**D**) TBAR levels in gastrocnemius muscle to TBARs levels in gastrocnemius muscle. (**E**) Changes in body weight, ovarian fat mass and retroperitoneal fat pad mass after PM_2.5_ exposure; representative data are shown (n = 6). Data are presented as mean ± standard deviation. Data were analyzed using two-sided unpaired Student’s t-tests or non-parametric test (* p < 0.05; ** p < 0.01; *** p < 0.001; ns, not significant, p>0.05).

The size of soleus muscle fibers exposed to PM_2.5_ was restored in old female mice, along with enhanced levels of muscle grow-related factors (myostatin and MyoG) and Pax-7 (Figs. 7A and B), which are linked to adult satellite cells and are key to myogenesis modulation. Mitochondria in the soleus muscle fibers of old PM_2.5_-exposed female mice were smaller (Fig. 7C) and significantly elevated expression levels of ATPsyn were observed (Fig. 7D). Among mitochondrial fission-associated factors, a significant increase in Fis-1 led to excessive mitochondrial fission despite down-regulation of DRP-1 expression (Fig. 7E). In contrast, among mitochondrial fusion-associated factors, only expression of the mitochondrial endo-connecting protein OPA-1 was increased, whereas expression of the more important mitochondrial outer and inner membrane-fusion protein Mfn-1/2 was not significantly increased (Fig. 7E), resulting in a kinetic imbalance in which mitochondrial size could not be restored. Compared with old control female mice, old PM_2.5_-exposed female mice exhibited defects in lipid metabolism with significantly lower phosphorylation of ACC (Fig. 8A), with a statistically activated expression level of GLUT-4 of glucose metabolism (Fig. 8B). As shown in Figs. 8C and D, old PM_2.5_-exposed female mice simultaneously underwent a redox reaction with enhanced levels of antioxidant-related factors such as catalase and GPx-4 and lipid oxidation-related factors such as TBARs. Unlike the gastrocnemius muscle fibers of old PM_2.5_-exposed male mice, those of old PM_2.5_-exposed female mice were inflamed, and significantly enhanced expression of NF-κB (Fig. 7B) was evident. Old PM_2.5_-exposed female also showed evidence of an increase in their weight and fat mass compared with corresponding female control mice (Fig. 8E). Taken together, these findings indicate that initial exposure to PM_2.5_ has long-term negative impacts on skeletal muscle modulation until aging, particularly in female mice. They also suggest that female mice exposed to PM_2.5_ at a young age are more susceptible to metabolic disorders compared with male mice exposed later in life.

## DISCUSSION

The skeletal muscular system, one of the largest metabolic organs in the body, plays an important role in physical movement and overall health. A direct connection between healthy skeletal muscle and the quality of life has been well-documented [41]. While PM2.5 exposure is known to provoke oxidative stress and inflammation in various tissues [42, 43], its long-term effects on skeletal muscle modulation remain inadequately understood. In addition, although stress exerts a differential impact according to gender [44], the gender-dependent mechanism through which PM_2.5_ affects the skeletal muscle system remains unclear.

**Figure 9.**
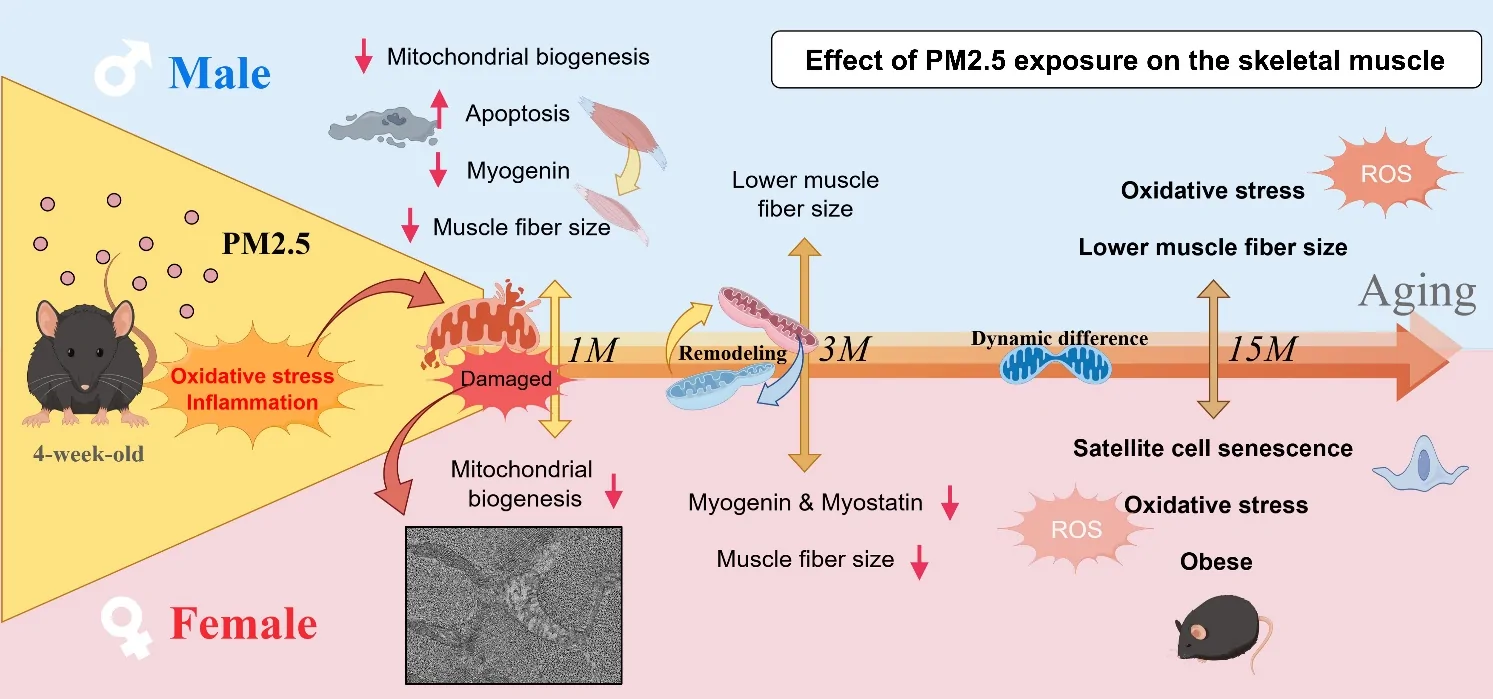
Schematic illustration of the exposure of young mice to atmospherically relevant PM_2.5_ has sex-dependent long-lasting impacts on the skeletal muscle system (By Figdraw, SOASOe9909).

This study demonstrates that atmospherically relevant PM_2.5_ exposure can have lasting, sex-dependent effects on skeletal muscle growth, mitochondrial integrity, and metabolic health in young mice (Figs. 1-8). Our findings indicate that PM_2.5_-induced mitochondrial dysfunction and muscle dysregulation in early life persist into aging, with female mice experiencing more severe effects than males (Fig. 9). These results underscore the chronic nature of PM_2.5_-induced oxidative stress, which impairs mitochondrial biogenesis and function, contributing to muscle degeneration and potential metabolic dysregulation. This adverse impact appears to be amplified by sex-dependent mechanisms, likely influenced by hormonal differences.

To clarify these mechanisms, future research should investigate molecular pathways relevant to muscle and mitochondrial function, including reactive oxygen species production, muscle protein turnover, and lipid accumulation, as well as the role of sex hormones in PM_2.5_-induced oxidative stress. Estrogen, for instance, has been shown to affect inflammatory and oxidative stress pathways in response to pollutants, potentially exacerbating mitochondrial and muscle cell damage in female mice [45]. Estrogen’s influence on mitochondrial biogenesis and electron transport efficiency may contribute to observed sex differences in mitochondrial damage and recovery [46-48]. Research has shown that estrogen can enhance inflammatory responses to environmental stressors like PM_2.5_, influencing cytokine release and oxidative stress markers, which could heighten cellular stress in response to pollutants [45]. Additionally, estrogen affects mitochondrial health by modulating the expression of proteins like PGC-1α, which regulates oxidative phosphorylation and energy homeostasis. Reduced mitochondrial function observed in female mice may be partly due to estrogen-mediated shifts that increase susceptibility to PM_2.5_-induced oxidative damage, impairing skeletal muscle function [46, 49].

The sex-specific responses identified here also relate to metabolic adaptations to PM_2.5_ exposure. Male mice exhibited early inhibition of muscle growth factors such as MyoG, impairing fiber development and possibly indicating a shift toward apoptotic pathways in response to oxidative stress (Fig. S2A) [50, 51]. Conversely, female mice displayed delayed muscle degeneration despite early mitochondrial damage, with compensatory changes in myostatin and MyoG expression emerging at 3 months post-exposure (Fig. 4B). This delayed fiber atrophy suggests that estrogen may initially buffer PM_2.5_-induced muscle damage, though this protective effect diminishes with age as estrogen levels decline, leading to compensatory mitochondrial dysregulation, redox imbalance, and an increase in the senescence state of satellite stem cells (Fig. S3B) in aged female muscles. Research supports the influence of estrogen fluctuations on mitochondrial dynamics, lipid oxidation, and glucose metabolism, which may drive the observed sex differences in PM_2.5_ impact [52-55].

One limitation of this study is the absence of direct PM_2.5_ measurements in plasma, which would establish the bioavailability and systemic impact of PM_2.5_. Additional analyses of specific markers of mitochondrial and muscle function-such as detailed ROS measurements, lipid oxidation markers, and satellite cell activation-would help clarify the precise pathways by which PM_2.5_ disrupts muscle homeostasis [56, 57]. Understanding these mechanisms could reveal therapeutic targets for mitigating PM_2.5_-induced muscle degeneration, particularly in females who may face an increased risk of muscle-related metabolic disorders due to prolonged exposure.

While our findings provide a foundational understanding of PM_2.5_’s chronic effects on muscle physiology, further research should examine molecular pathways related to mitochondrial fusion, fission, and autophagy to clarify the observed dynamics. Studies on protein turnover, lipid metabolism, and inflammation markers would enhance our understanding of PM_2.5_’s systemic impact. It is also essential to acknowledge that limitations in exploring muscle function, protein degradation pathways, and inflammatory markers may introduce bias, potentially limiting the generalizability of these findings to other animal models and human health implications. In conclusion, this study highlights the need for longitudinal research on the effects of PM_2.5_ exposure on skeletal muscle, especially to assess the permanency or reversibility of PM_2.5_-induced changes. Research into potential interventions, such as antioxidants or mitochondrial stabilizers, could provide insights into strategies to combat PM_2.5_-associated muscle degradation. Given the significant public health implications of air pollution, particularly for susceptible populations, these findings emphasize the need for understanding and control of sex-based disparities in PM_2.5_ impacts on muscle health.

## Supplementary Materials

The Supplementary data can be found online at: www.aginganddisease.org/EN/10.14336/AD.2024.1047.
