# Supplementary material for: Exposure of Young Mice to Atmospherically Relevant PM2.5 Has Sex-Dependent Long-Lasting Impacts on the Skeletal Muscle System
Source: Aging Dis. 2024 Dec 2;16(6):3690–705. doi: 10.14336/AD.2024.1047 (PMC12539521; doi:10.14336/AD.2024.1047)
Supplement: Supplementary file 1 — The Supplementary data can be found online at: www.aginganddisease.org/EN/10.14336/AD.2024.1047. [file AD-16-6-3690-s.pdf]

SUPPLEMENTARY DATA

Exposure of Young Mice to Atmospherically Relevant $PM_{2.5}$ Has Sex-Dependent Long-Lasting Impacts on the Skeletal Muscle System

Wenduo Liu, Zilin Wang, Min-Hye Kim, Yu Gu, Hyun-Jaung Sim, Jeong-Chae Lee, Sung-Ho Kook, Sang Hyun Kim

© 2024. Liu W et al. Published online at http://www.aginganddisease.org/EN/10.14336/AD.2024.1047

SUPPLEMENTARY DATA

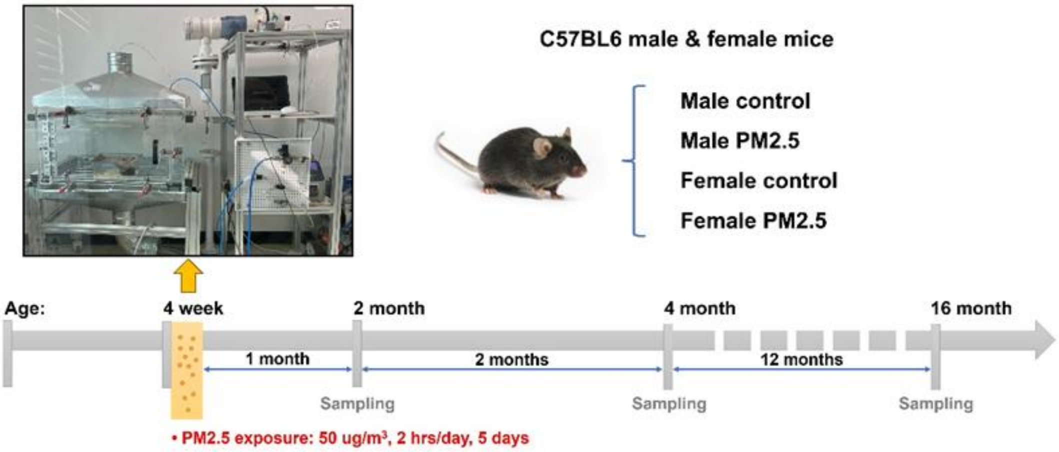

Supplementary Figure 1. Schematic of the experimental design, with groups divided and the time of sampling. A total of 72 female and male mice were randomly divided into a male-normal group (n = 18), a male $PM_{2.5}$-exposed group (n = 18), a female normal group (n = 18) and a female $PM_{2.5}$-exposed group (n=18). The $PM_{2.5}$-exposed group completed treatment at week 5 and were sampled 1 month, 3 months, and 15 months after $PM_{2.5}$ exposure.

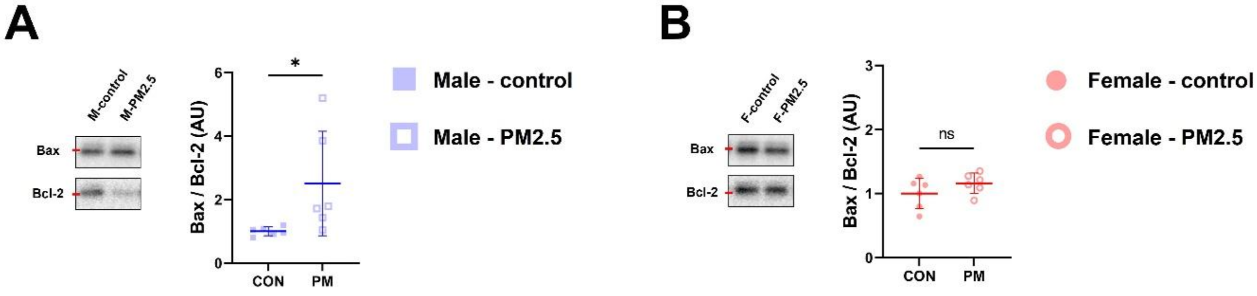

Supplementary Figure 2. Effect of $PM_{2.5}$ on apoptotic cell death in skeletal muscle fibers at 1-month post-exposure. **A**) Expression ratio of Bax/Bcl-2 in male mouse gastrocnemius muscle; representative data are shown (n = 6). **B**) Expression ratio of Bax/Bcl-2 in female mouse gastrocnemius muscle; representative data are shown (n = 6). Data are presented as mean ± standard deviation. Data were analyzed by two-sided unpaired Student's t-tests (* $p < 0.05$; ** $p < 0.01$; *** $p < 0.001$; ns, not significant, $p > 0.05$).

© 2024. Liu W et al. Published online at http://www.aginganddisease.org/EN/10.14336/AD.2024.1047

SUPPLEMENTARY DATA

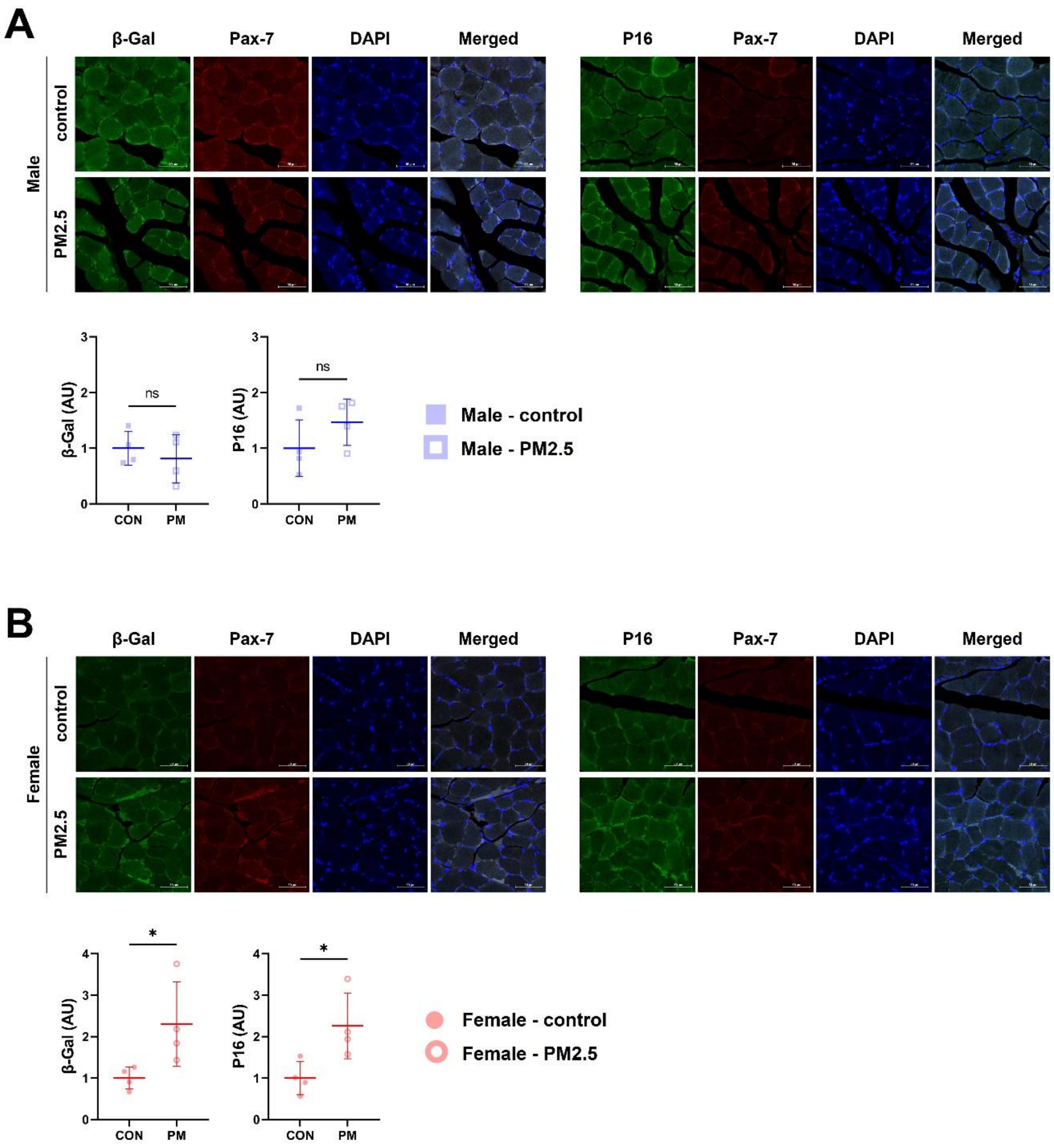

Supplementary Figure 3. Effect of $PM_{2.5}$ on senescence of satellite cells in skeletal muscle fibers at 15-month post-exposure. A) Expression levels of Pax-7/β-galactosidase and Pax-7/p16 were measured in male mouse tibialis anterior muscles by immunofluorescence; representative data are shown (n = 4). **B**) Expression levels of Pax-7/β-galactosidase and Pax-7/p16 were measured in female mouse tibialis anterior muscles by immunofluorescence; representative data are shown (n = 4). Data are presented as mean ± standard deviation. Data were analyzed by non-parametric test (* $p < 0.05$; ** $p < 0.01$; *** $p < 0.001$; ns, not significant, $p > 0.05$).

© 2024. Liu W et al. Published online at http://www.aginganddisease.org/EN/10.14336/AD.2024.1047

SUPPLEMENTARY DATA

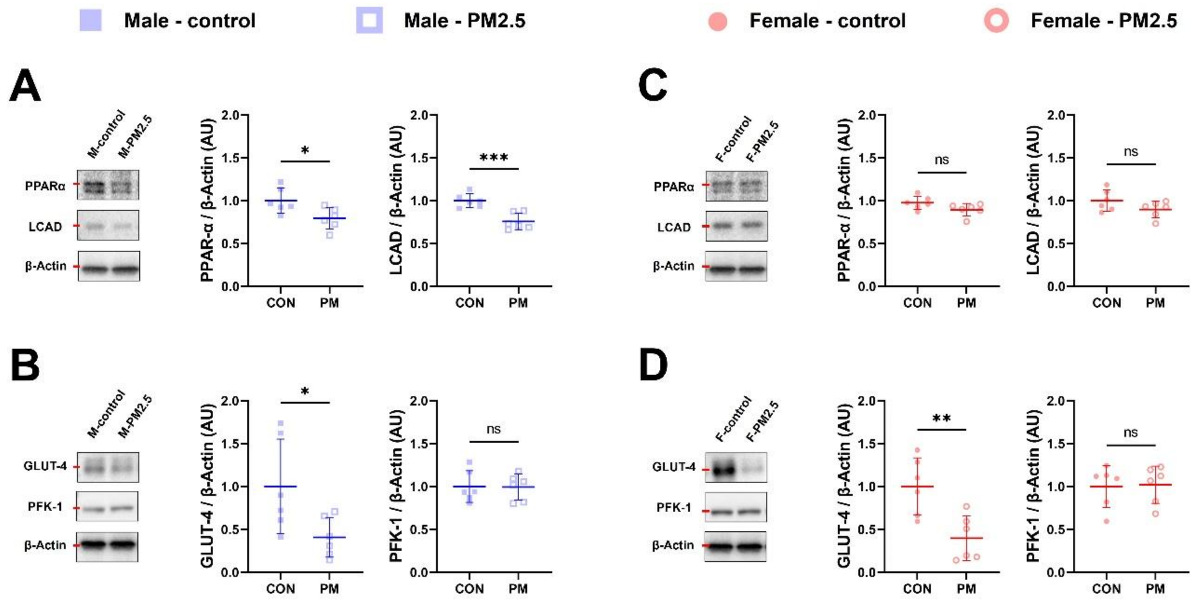

Supplementary Figure 4. Effects of $PM_{2.5}$ on glucose- and lipid metabolism-related factors in skeletal muscle fibers at 1-month post-exposure. A) Expression levels of PPAR-α and LCAD in male mouse gastrocnemius muscle; representative data are shown (n = 6). **B**) Expression levels of GLUT-4 and PFK-1 in male mouse gastrocnemius muscle; representative data are shown (n = 6). **C**) Expression levels of PPAR-α and LCAD in female mouse gastrocnemius muscle; representative data are shown (n = 6). **D**) Expression levels of GLUT-4 and PFK-1 in female mouse gastrocnemius muscle; representative data are shown (n = 6). Data are presented as mean ± standard deviation. Data were analyzed by two-sided unpaired Student's t-tests (* $p < 0.05$; ** $p < 0.01$; *** $p < 0.001$; ns, not significant, $p > 0.05$).

© 2024. Liu W et al. Published online at http://www.aginganddisease.org/EN/10.14336/AD.2024.1047

SUPPLEMENTARY DATA

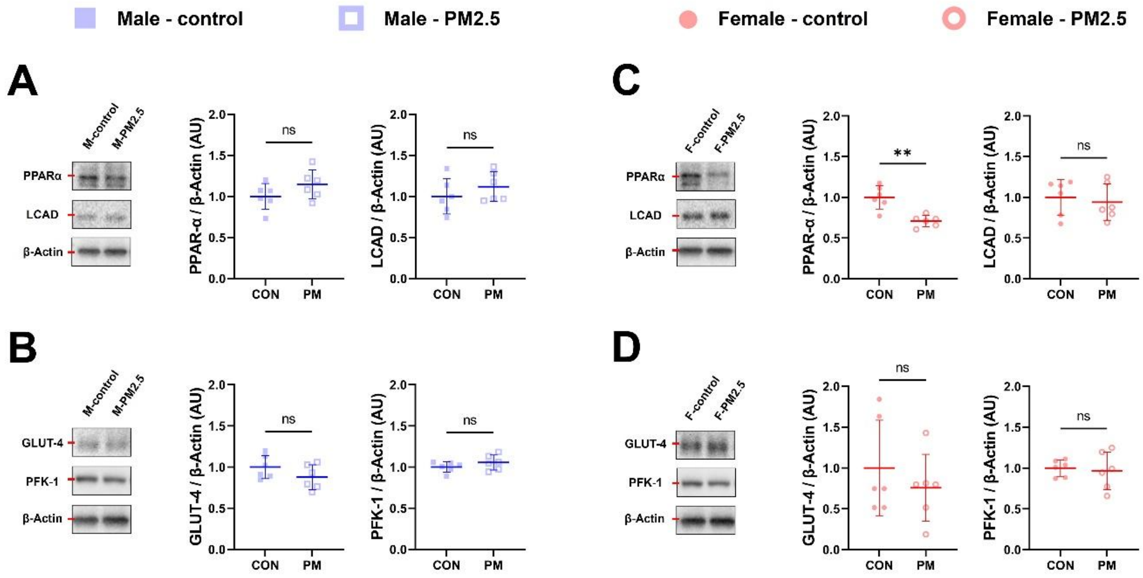

Supplementary Figure 5. Effects of $PM_{2.5}$ on glucose- and lipid metabolism-related factors in skeletal muscle fibers at 3-month post-exposure. A) Expression levels of PPAR-α and LCAD in male mouse gastrocnemius muscle; representative data are shown (n = 6). **B**) Expression levels of GLUT-4 and PFK-1 in male mouse gastrocnemius muscle; representative data are shown (n = 6). **C**) Expression levels of PPAR-α and LCAD in female mouse gastrocnemius muscle; representative data are shown (n = 6). **D**) Expression levels of GLUT-4 and PFK-1 in female mouse gastrocnemius muscle; representative data are shown (n = 6). Data are presented as mean ± standard deviation. Data were analyzed by two-sided unpaired Student's t-tests (* $p < 0.05$; ** $p < 0.01$; *** $p < 0.001$; ns, not significant, $p > 0.05$).

© 2024. Liu W et al. Published online at http://www.aginganddisease.org/EN/10.14336/AD.2024.1047

SUPPLEMENTARY DATA

Supplementary Table 1. List of the chemical compositions, formula, and dry mass fractions of organic and inorganic species used in this study.

Functional Group	Components	Formula	Density (g/cm^3) at 295 K*	Dry mass fraction (%)
Monocarboxylic acid	Acetate	$C_2H_3O_2$	1.05	6.25
Dicarboxylic acid	Oxalic acid	$C_2H_2O_4$	1.90	6.25
	Malonic acid	$C_3H_4O_4$	1.62	6.25
	Glutaric acid	$C_5H_8O_4$	1.35	6.25
Polyols	Glycerol	$C_3H_8O_3$	1.26	6.25
Sugars	Sucrose	$C_{12}H_{22}O_{11}$	1.59	6.25
Aromatics	2,5-Dihydroxybenzoic acid	$C_7H_6O_3$	1.55	6.25
Amino acid	Glycine	$C_2H_5O_2N$	1.61	6.25
Inorganic salts	Ammonium sulfate	$(NH_4)_2SO_4$	1.77	25
	Ammonium nitrate	NH_4NO_3	1.72	25

* Values of measured densities are from www.chemicalbook.com

Supplementary Table 2. Mean PM concentration in the exposure chamber.

Method	Particle counter (μg/m^{-3})
Batch1	57.6± 8.9
Batch2	51.9 ± 7.3
Batch3	43.9 ± 13.6
Batch4	49.6 ± 9.6
Batch5	51.4 ± 12.6

© 2024. Liu W et al. Published online at http://www.aginganddisease.org/EN/10.14336/AD.2024.1047
